# Precision of a Novel Craniofacial Surgical Navigation System Based on Augmented Reality Using an Occlusal Splint as a Registration Strategy

**DOI:** 10.1038/s41598-018-36457-2

**Published:** 2019-01-24

**Authors:** Taoran Jiang, Ming Zhu, Gang Chai, Qingfeng Li

**Affiliations:** 1 Department of Plastic and Reconstructive Surgery, Shanghai 9th People’s Hospital, Shanghai Jiao Tong University School of Medicine, Zhizaoju Road 639, Shanghai, 200011 People’s Republic of China; 2 Department of Plastic and Reconstructive Surgery, Zhongshan Hospital, Fudan University, No. 180 Feng Lin Road, Shanghai, 200032 People’s Republic of China

## Abstract

The authors have developed a novel augmented reality (AR)-based navigation system (NS) for craniofacial surgery. In this study, the authors aimed to measure the precision of the system and further analyze the primary influencing factors of the precision. The drilling of holes into the mandibles of ten beagle dogs was performed under the AR-based NS, and the precision was analyzed by comparing the deviation between the preoperational plan and the surgical outcome. The AR-based NS was successfully applied to quickly and precisely drill holes in the mandibles. The mean positional deviation between the preoperative design and intraoperative navigation was 1.29 ± 0.70 mm for the entry points and 2.47 ± 0.66 mm for the end points, and the angular deviation was 1.32° ± 1.17°. The precision linearly decreased with the distance from the marker. In conclusion, the precision of this system could satisfy clinical requirements, and this system may serve as a helpful tool for improving the precision in craniofacial surgery.

## Introduction

Augmented reality (AR) is a computer technology that can provide virtual information to real environments and improve visual comprehension. This technology has been used in a variety of applications, especially in navigation systems for surgery^1–3^. The AR-based navigation system (NS) superimposes a virtual 3-dimensional inner structure that was reconstructed from the patients’ computed tomography (CT) or magnetic resonance imaging (MRI) data onto the real surgical field. This advanced technology can help prevent surgeons from performing operations only according to their experience and enable more accurate surgeries.

To achieve an esthetic, symmetrical appearance and avoid injuring the blood vessels and nerves in the head and face, the bone resection and reconstruction during craniofacial surgery require a high degree of precision and have a narrow margin of error. Therefore, we developed an AR-based NS for craniofacial surgery that used an occlusal splint as the registration strategy to minimize the risks and improve the precision of the surgery. This system could be applied in treating orbital hypertelorism, hemifacial microsomia, mandibular angle split osteotomy and other abnormalities^4–6^. Because the precision of the system will have a great impact on the surgical outcome, we designed this study to measure the precision of the system and evaluate the primary influential factors.

## Result

**Figure 1:**
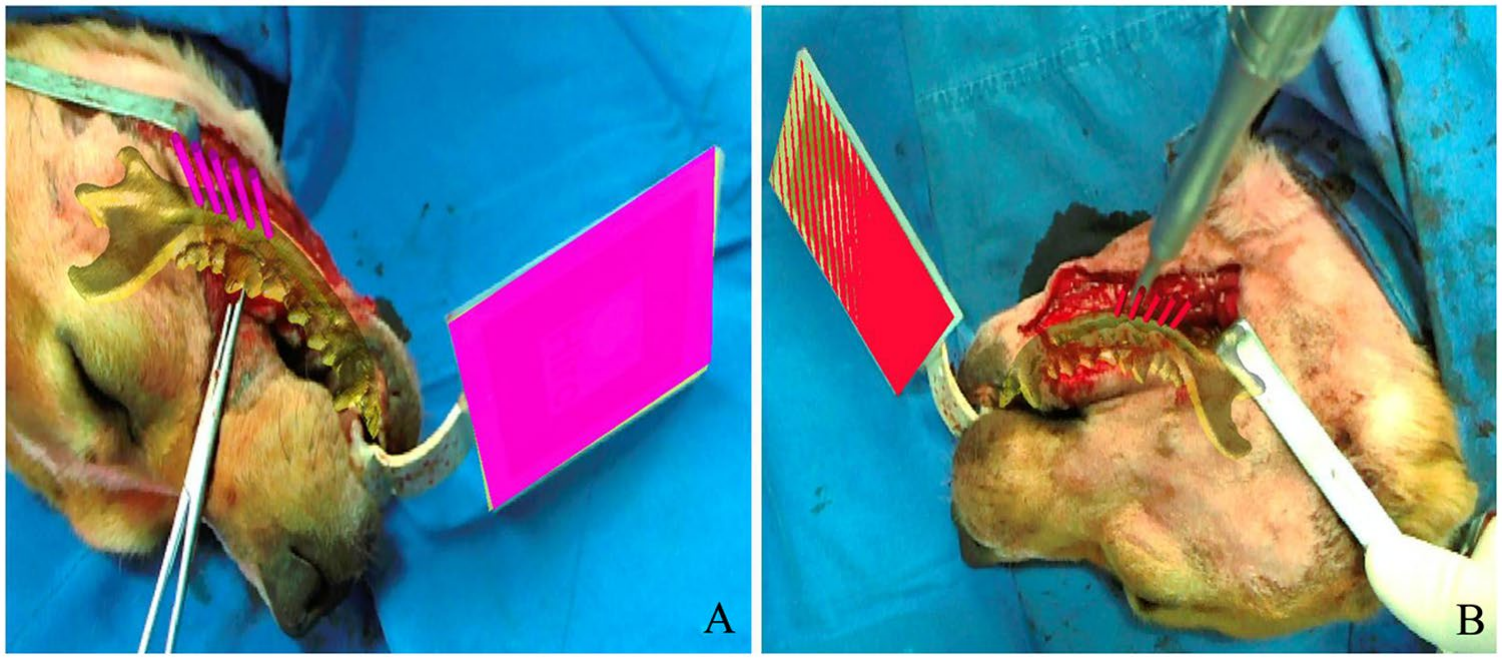
Photographs taken during operation. (**A**) Virtual 3D model and guidance information were projected onto the real world though the AR-based NS. (**B**) Drilling the holes according to the navigation.

The AR-based NS was successfully applied for precisely controlling the drilling of holes in the mandibles for mandibular angle split osteotomy in ten randomly selected beagle dogs. Because the fiducial marker was firmly fixed to the mandible though the occlusal splint, the relationship between the marker and mandible was retained throughout the operation. The NS achieves the “seamless” integration of the virtual and real situations, and the virtual image can stably track with the mandible. Under the guidance of the NS, the surgeon felt more confident in performing the drilling. The mean time required for drilling five holes with the NS was 208.0 ± 11.9 s, which was much shorter than that without the NS (365.7 ± 9.6 s) (Fig. 1, Table 1). All surgeries for the ten dogs were performed by the same surgeon.

**Table 1:** Time required for drilling holes with or without NS.

	Time required for drilling holes with the NS(s)	Time required for drilling holes without the NS(s)	
Maximum	222.5	380.2	
Minimum	196.3	353.4	
Mean	208.0 ± 11.9	365.7 ± 9.6	P < 0.05

By comparing the positions and angles between the actual holes and preoperative designs, the deviations of the position and angle of the preoperative design and intraoperative navigation could be confirmed. The mean distance deviation of the entry points between the preoperative design and intraoperative navigation was 1.29 ± 0.70 mm (range from 0.31 to 1.94 mm), and the deviation was 2.47 ± 0.66 mm for the end points (range from 1.91 to 3.55 mm). The angular deviation ranged from 0.97° to 2.89°, and the mean error was 1.32° ± 1.17° (Table 2).

**Table 2:** Details of the deviation between the preoperative design and navigation result.

	Positional deviation at entry point (mm)	Positional deviation at end point (mm)	Angular deviation
Maximum	1.94	3.55	2.02°
Minimum	0.31	1.91	0.97°
Mean	1.29 ± 0.70	2.47 ± 0.66	1.32° ± 1.17°

## Discussion

In past decades, computer-aided navigation systems have undergone tremendous development from virtual reality (VR)-based NSs to augmented reality-based NSs. Compared with VR-based navigation, AR-based navigation can seamlessly combine virtual and real information to generate a “see-through” image instead of the totally immersive mode in VR. When wearing a head-mounted display (HMD), the virtual images could overlay onto the wearer’s field of view, and the wearer has no need to learn new hand-eye coordination skills. Because of these advantages of AR, a system based on AR seems more appropriate for surgical navigation. Under the guidance of a AR-based NS, bone resections and reconstructions in anatomically distorted or complex areas, such as the head and neck region, could be more effective and precise with reduced risk of nerve and vessel injury^7,8^. The time required for the operations appeared to be much less with the NS. In our study, the time required for drilling holes was decreased by more than half with the NS, from 365.7s to 208.0s. However, only four comparative experiments have been completed, and all surgeries were performed by the same surgeon, so it is difficult to reach the conclusion that all surgeries with the NS would be quicker than those without the NS. In a further study, robust data will be collected to reach a final conclusion. However, with the guidance of the NS, the surgeon performed the drilling of holes without hesitation and did not need to repeatedly confirm the locations of holes, which may result in a quicker surgery.

Many performance parameters could be used to evaluate the feasibility of an AR system, such as the precision, resolution, response time and robustness^9^. However, for a surgical navigation system, the precision is the most important parameter, in that it could directly influence the result of the surgery. In our prior clinical studies^4–6^, we have verified the feasibility and effectiveness of our AR-based NS, but during the clinical application we found that the system had position errors. Hence, we designed this study to analyze the precision of the NS for craniofacial surgery. Since the movement of the bone mass after osteotomy could increase the error, we only drilled holes in the mandible of an animal model to exclude this error. The use of an animal model could mimic each step in the clinical application to obtain the overall deviation. According to our result, the mean distance error was 1.29 mm at the entry point and 2.47 mm at the end point, while the mean angular deviation was 1.32°.

The deviation of an ideal surgical navigation should be less than 1.5 mm^10^. However, this standard is difficult to reach. The more complicated the surgical procedure is, the greater the error is. Vigh *et al*. reported the accuracy of an AR navigation system for drilling holes in the mandible to have mean position errors of 1.24 mm at the entry point and 2.68 mm at the end point and to have a mean angular deviation of 4.68°^11^. This result was similar to that of our study, but the deviation of the end point remained larger than the standard. In the study by Wang *et al*., the mean position errors of an AR-based navigation system for sacroiliac screw insertion were 2.7 mm at the entry point and 3.7 mm at the end point, while the mean angular deviation was 2.9°^12^. The deviation was much higher than in our and Vigh’s studies due to the more complicated procedure. In the study of Franz *et al*., under the guidance of an optical navigation system, the mean discrepancy between the positions of the planned and real osteotomy lines was 0.044 mm. To reflect the real range of the variability in the measured discrepancies, the authors also calculated the interval of possible positional differences, which ranged from −1.500 to 1.589 mm. This result met the navigation requirement, but in this study, the authors only performed the simplest procedure, namely, marking the osteotomy line^13^.

The overall deviation depends on the compounding of errors produced from the very beginning, during the CT/MRI data acquisition, through the end of the surgical procedure. This includes technical (hardware and software errors), imaging, registration, tracking and human errors^14^. Among these, the registration process, which is responsible for the accurate linking of the virtual image to the surgical site, has the greatest impact on the precision of the navigation system because it has direct effects on the precision of all subsequent navigation tasks^15–22^. Therefore, researchers have extensively investigated the topic of the registration, and many studies have compared the accuracy of intraoperative navigation in craniofacial surgery performed using different registration methods. According to the characteristics of craniofacial surgery, the following four registration methods could be used: bone implants (screws), occlusal splint fitted to the teeth, anatomic landmarks, and laser surface scanning. Among these methods, screws can achieve the highest precision; however, this invasive method cannot be used for most plastic surgery patients. Anatomic landmarks and laser surface scanning are comparatively practical to use because of their noninvasive nature and lack of requirement of any reference markers. However, the soft tissue could shift and deform during the operation, which will greatly affect the precision of these two methods. Occlusal splints provide a noninvasive, highly accurate registration method that is steadily fitted to stable bony landmark-dental cups^23–25^. Due to its advantages, this method has been widely used in cranio-maxillo-facial surgical navigation. According to the study of Venosta *et al*.^26^, a splint of a considerable size could be an obstacle during periorbital and maxillofacial surgery and influence the effect of the navigation. We thus used a narrow-edged occlusal splint (from lateral to lateral incisors) to fix the fiducial marker in our study to reduce its interference with the intraoral operation. In our clinical application^4,5^, such a narrow-edged occlusal splint was stable and reliable for the registration.

By applying occlusal splints as the registration method, the precision of our AR-based craniofacial surgical navigation system could meet the clinical requirements. However, the deviation of the end point was larger than that of the entry point, and this phenomenon was also observed in the study by Vigh *et al*. Based on our experience, human error is the main cause of this phenomenon. The more skilled the surgeon is, the lower the error is. In our study, the surgeon had more than twenty years of experience in craniofacial surgery, while in Vigh’s study, the surgeons had only ten years of experience or even no surgical experience. This may explain why the end point error and the angle error of Vigh’s study were higher than ours.

**Figure 2:**
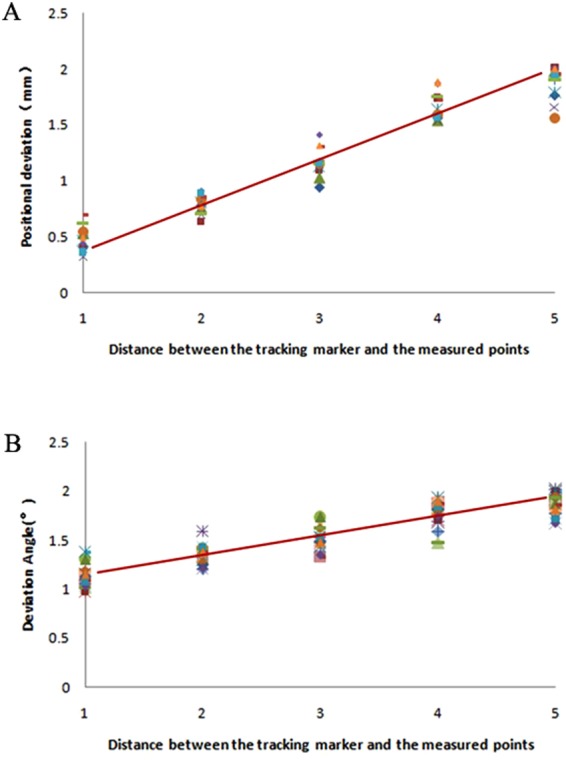
Scatter graphs representing the relationship between the deviation (**A**) position deviation, (**B**) angle deviation) and the distance between the tracking marker and the measured points.

With the further statistical analysis of the experimental data, we found that the closer (farther) the distance from the marker, the smaller (larger) the positional deviation, and the angular error had the same trend (Fig. 2). This phenomenon was prominent when registering via bone screws and an occlusal splint. This finding suggests that the reference marker should not be too far from the surgical area; otherwise, it will affect the precision of the navigation. The location of the marker should be as close as possible to the surgical area without affecting the operation. An occlusal splint may be sufficient for the navigation of maxillary and mandible surgery, while for skull base and fronto-orbital surgery, it should be combined with other registration methods, such as bone implants inserted on the orbital rim, to obtain an ideal precision. However, for some patients who cannot undergo an invasive registration method, the precision of registering via an occlusal splint alone could still meet the clinical requirement^5^.

## Conclusion

We demonstrated in our previous studies that our AR-based NS, which used an occlusal splint as a registration strategy, was feasible for craniofacial surgery. Additionally, in this study, we demonstrated that the precision of the system could satisfy the clinical requirements. However, the precision deterioration over a distance is prominent when registering via the occlusal splint, and it is necessary to select appropriate locations for reference markers according to the surgical area.

## Methods

To analyze the precision of this AR-based navigation system, ten beagle dogs were randomly chosen for use as our model. The drilling of the mandibles of these beagle dogs was performed under the AR-based NS. A three-dimensional (3D) CT scan was performed pre- and postoperatively to gather information to generate guiding information for the NS and measure the precision, respectively. All animal experiments were approved by the Institutional Animal Care and Use Committee (IACUC) of Shanghai Jiao Tong University School of Medicine and were in compliance with relevant guidelines and regulations.

### Preoperative preparation

**Figure 3:**
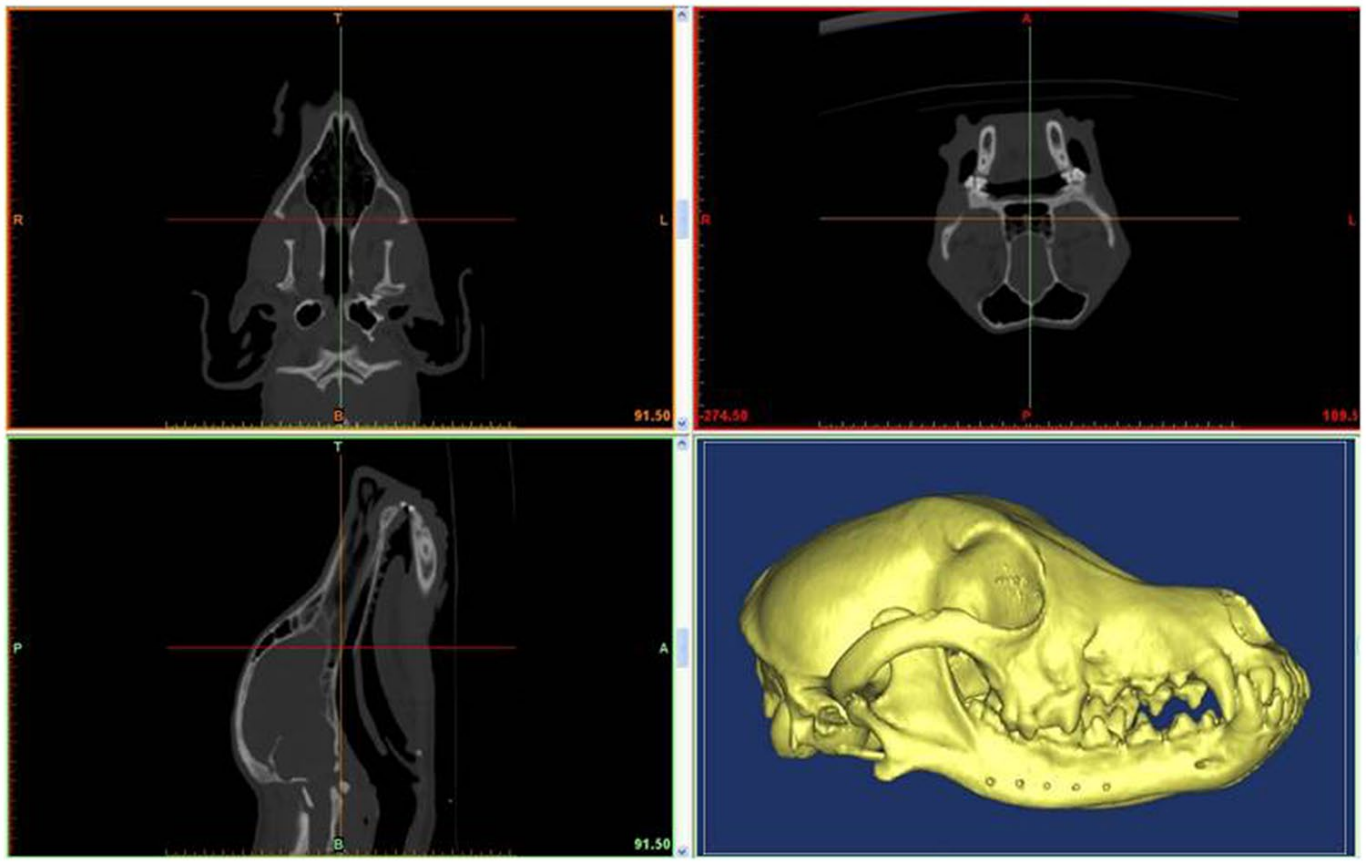
Reconstructed 3D model of mandible and preoperative design of drilling holes.

Before the surgery, thin-cut (1.25-mm) CT scans of the mandibles were performed, and the data were imported into Mimics 15.0 software (Materialise, Leuven, Belgium) to reconstruct a 3D digital model and design the surgical procedure. As indicated in the 3D digital model, the virtual images of the mandible, critical nerves, and soft tissues can be clearly observed. With these data, the cutting plane was designed accurately and individually to avoid critical nerves. Additionally, five drill holes were designed in the cutting plane (Fig. 3).

**Figure 4:**
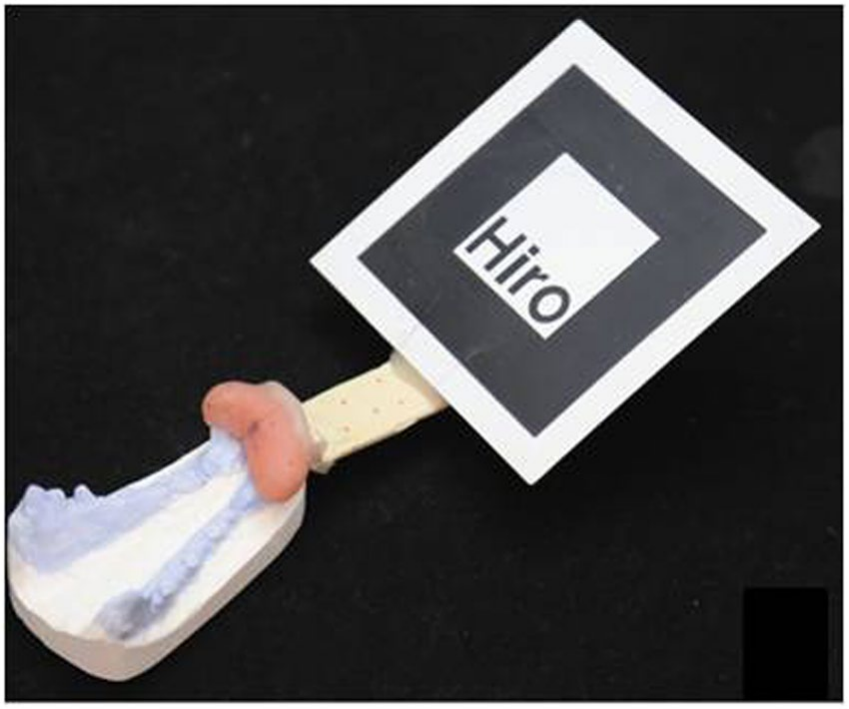
Occlusal splint with marker (OSM) stably clutching the dental cast.

After the navigation information was prepared, we began to register the virtual image with the real world. A fiducial marker was considered as a reference between the virtual image data and real world to maintain a permanent, firm relationship with the mandible. For that marker, we used occlusal splints to stabilize the relationship between the marker and skull. Specifically, a submaxillary dental cast was prepared for each dog, and the occlusal splint was created according to the central incisor and lateral incisor on both sides of the dental cast. Then, the fiducial marker was fixed to the occlusal splint to form the occlusal splint and marker (OSM) complex. In this way, the marker and mandible were kept in a permanent relationship during the operation through constant occlusion (Fig. 4). After 3D laser scanning was performed for the dental cast and OSM, the digital data were obtained. The 3D images of the OSM and mandible were integrated using engineering software (Rapidform2006, Inus Technology, Seoul, Korea) according to three or four dental cusps. As a result, the virtual image included the cutting plane and drill holes on the mandible as well as the fiducial marker and occlusal splint.

The software ARToolKit (ARToolworks, Seattle, WA, USA) can project a three-dimensional virtual model to the real world by recognizing a marker, and the virtual model was automatically projected to the center of the marker. To register the virtual model with an actual position, the position and orientation of the virtual image were adjusted using 3ds Max software (Autodesk, San Rafael, CA, USA) to ascertain that the central point of the marker overlapped with the origin of the coordinate system.

### Intraoperative navigation and postoperative analysis of deviation between the preoperative design and actual surgical outcome

**Figure 5:**
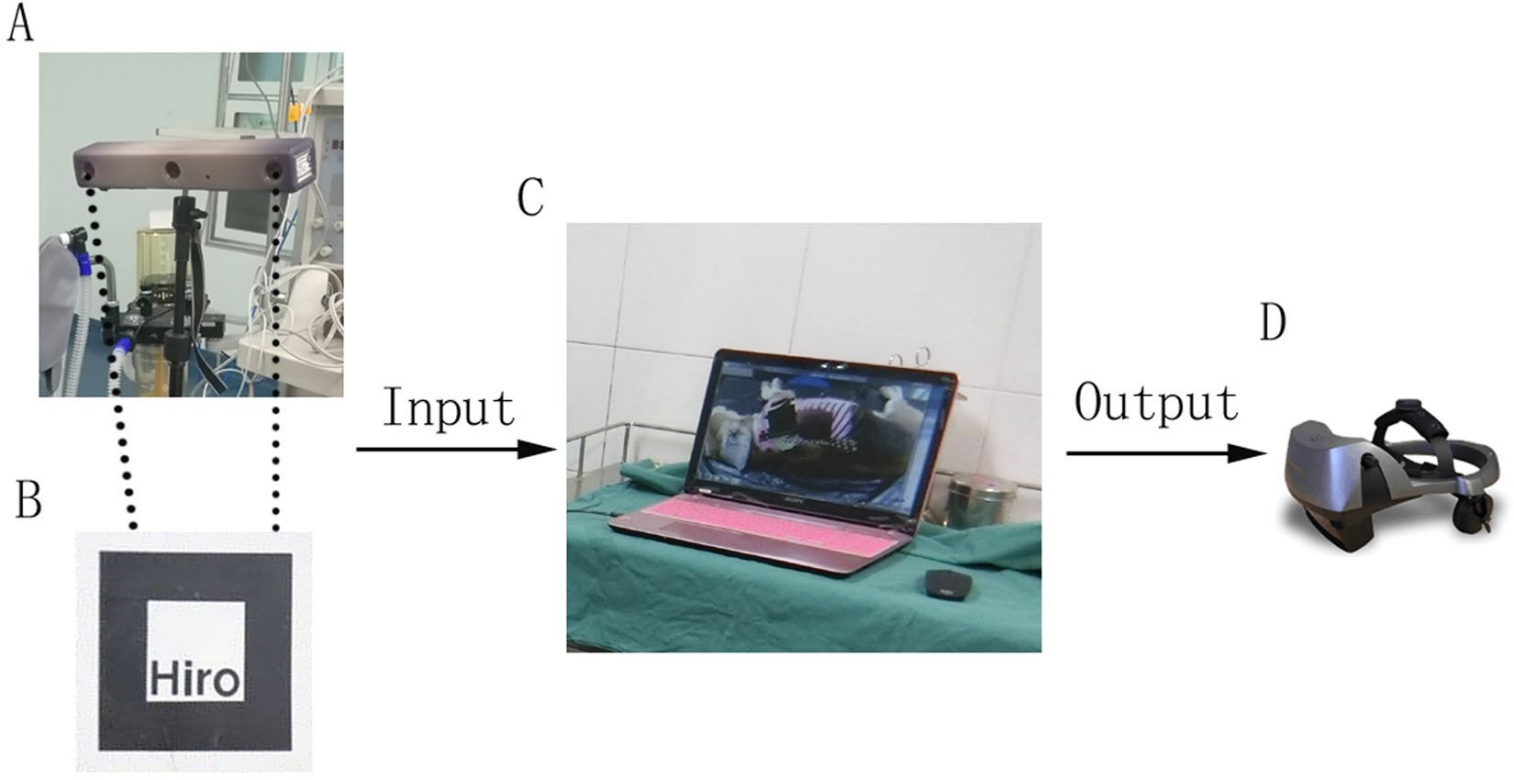
Set-up of the AR-based NS. (**A**) Three-dimensional digital camera. (**B**) Marker. (**C**) Computer workstation. (**D**) Helmet-mounted display (HMD). The 3D camera inputs the view of the operating room into the computer workstation. As soon as ARToolkit recognized the marker, the seamless integration pictures of the virtual model and real situation were output to the HMD in real time.

The components of the system include a three-dimensional digital camera, a helmet-mounted display (nVisor ST60, NVIS Company, USA), a high-definition monitor and a computer workstation (Fig. 5). The 3D camera was endowed with 2 cameras to merge 3D images and provided a view of the operation field. As soon as the fiducial marker was recognized, the software ARToolkit projected the 3D virtual image to the real world according to the center of the marker. Then, the “seamless” integration images of the virtual and real situations were exported to the helmet-mounted display for the surgeon, as well as the high-definition monitor for the assistants. The surgeon drilled five holes on each side of the mandible of six beagle dogs according to the preoperative design in the virtual image. For another four beagle dogs, the surgeon drilled holes of the mandible according to the preoperative design with the navigation on one side and without the navigation on the other side. The operative time on each side was recorded by a stopwatch.

**Figure 6:**
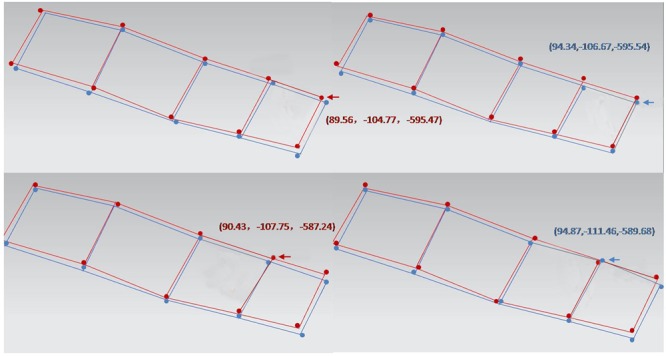
Comparison of postoperational drill hole with preoperational design: red points are the design drill holes, and blue points are the operative results. The distance between the red points and blue points is the positional deviation of the navigation, and the angle between the red lines and blue lines is the angular deviation.

**Figure 7:**
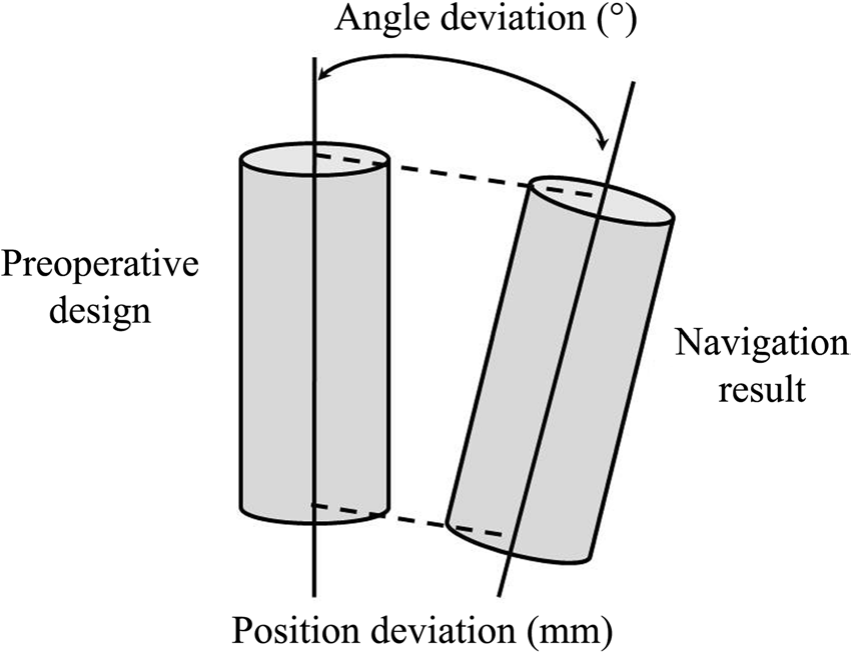
Sketch map of deviation measurement.

One week after the surgery, postoperative CT scanning was performed, and the data were imported into Mimics 15.0 to reconstruct a 3D digital model of the mandible. Based on that model, the actual holes drilled using the intraoperative navigation and their three-dimensional coordinates were identified. By a matrix transformation operation, the preoperative design and actual drill holes were mapped to the same coordinate system, and the positional and angular errors of each hole were calculated (Figs 6 and 7).

### Statistical analysis

Continuous variables were summarized by the mean and standard deviation. The times required for drilling five holes with or without NS were analyzed by Student’s t-test. A p-value less than 0.05 was considered as significantly different.

## Data Availability

Data are fully available through the corresponding author on reasonable request.
